# Revision of the Australasian genus *Pseudidarnes* Girault, 1927 (Hymenoptera, Agaonidae, Sycophaginae)

**DOI:** 10.3897/zookeys.404.7204

**Published:** 2014-04-22

**Authors:** Fernando Henrique Antoniolli Farache, Jean-Yves Rasplus

**Affiliations:** 1 PPG em Entomologia, Depto de Biologia/FFCLRP-USP Ribeirão Preto, SP, Brazil; 2 INRA, UMR 1062 CBGP Montferrier-sur-Lez, France

**Keywords:** Chalcidoidea, *Ficus*, Moraceae, non-pollinating fig wasp, gall maker

## Abstract

The species of *Pseudidarnes* are revised, and six species are described: *P. acaudus* Farache & Rasplus, **sp. n.**; *P. astridae* Farache & Rasplus, **sp. n.**; *P. badiogeminus* Farache & Rasplus, **sp. n.**; *P. cooki* Farache & Rasplus, **sp. n.**; *P. kjellbergi* Farache & Rasplus, **sp. n.**; *P. laevis* Farache & Rasplus, **sp. n.** *Pseudidarnes minerva* Girault, 1927 and *P. flavicollis* Bouček, 1988 are redescribed. A key to the species is provided as well as illustrations for all females and all known males (except the wingless male of *P. minerva*). We also provided further discussion on ecology, morphological patterns, and host taxonomy. Online dichotomous and multi-access interactive LUCID keys to all *Pseudidarnes* species are available at http://www.figweb.org/.

## Introduction

Sycophaginae is one of the six subfamilies of chalcid wasps that are strictly associated with *Ficus* syconia. A recent phylogenetic analysis of the Chalcidoidea recovered Agaoninae (fig pollinators) + Sycophaginae as a monophyletic group (Agaonidae), whereas all other fig wasp subfamilies were included in Pteromalidae (Heraty et al. 2013). Sycophaginae are associated with only two subgenera of fig trees (*Urostigma* and *Sycomorus*). Most of the species oviposit in figs (syconia) through the fig wall and are gall inducers or parasitoids of other fig wasps. However, some *Sycophaga* species are capable of entering the figs through a small pore (ostiole), as pollinating fig wasps do (Bouček 1988, 1993, Cruaud et al. 2011a, Cruaud et al. 2011b, Elias et al. 2008, Rasplus and Soldati 2005).

The Sycophaginae occur in all tropical regions of the world but are still poorly known, with about 60 described species and an estimated diversity of about 700 species (Cruaud et al. 2011b). There are five described genera of Sycophaginae, and at least one genus awaits description (Bouček 1988, Cruaud et al. 2011a, Cruaud et al. 2011b). Four of these genera occur in the Australasian region, namely *Pseudidarnes* Girault, 1927, *Eukoebelea* Ashmead, 1904, *Sycophaga* Westwood, 1840, and the undescribed genus (Ashmead 1904, Bouček 1988, Cruaud et al. 2011a, Cruaud et al. 2011b, Westwood 1840).

The phylogeny of the Sycophaginae recovered by the analysis of multiple genes (Cruaud et al. 2011a, Cruaud et al. 2011b) showed that the subfamily is subdivided into three main clades:

1) *Eukoebelea* (associated with Australasian *Malvanthera* fig trees) recovered as the sister lineage to all other genera.

2) A strongly supported clade of three genera, *Pseudidarnes* (*Malvanthera*) basal to *Anidarnes* Bouček, 1993 (associated with the New World *Americana* fig trees) plus the undescribed genus (associated with the Oriental *Conosycea* figs). A dichotomic key separating these three genera is provided by Farache et al. (2013).

3) A well-supported clade composed of two groups: *Sycophaga* (mostly associated with Australasian, oriental and afrotropical *Sycomorus* fig trees) and *Idarnes* Walker, 1843 (associated with the New World *Americana* figs).

The biogeography of the subfamily has been discussed by Cruaud et al. (2011a). *Pseudidarnes* is only known from the Australasian region and includes two described species: *Pseudidarnes minerva* Girault, 1927 from Australia, associated with *Ficus rubiginosa* (Girault 1927), and *Pseudidarnes flavicollis* Bouček, 1988, described from Papua New Guinea and reared from *Ficus xylosycia* (Bouček 1988), but never collected again. *Pseudidarnes* species are strictly associated with syconia of *Ficus* section *Malvanthera* Corner (subg. *Urostigma*). This section includes 23 fig species mostly occurring in Australia and New Guinea (incl. Bismarck Archipelago), reaching Sulawesi, the Vanuatu islands and New Caledonia (Dixon 2003, Rønsted et al. 2008). *Malvanthera* fig trees are pollinated by *Pleistodontes* Saunders wasps (Lopez-Vaamonde et al. 2002).

Among the guilds that constitute fig wasp communities, *Pseudidarnes* species belong to the “large gall inducers” (Cruaud et al. 2011b, Segar and Cook 2012, Segar et al. 2013). The non-pollinating fig wasps belonging to this guild are larger than the co-occurring pollinators, exhibit short and thick ovipositors, and oviposit through the syconium wall during the early development of the syconia before pollination (Cruaud et al. 2011b, Elias et al. 2008). Induction of large galls is usually correlated with small brood sizes, and their galls may occupy the whole syconium lumen (Bronstein 1999; Cook et al. 1997, West and Herre 1998). As other large gall inducers, *Pseudidarnes* species are usually rare in most host species and found in low abundance (0.1 ± 0.5, mean ± *SE*, insects per fig in *Ficus obliqua*) (Segar and Cook 2012).

*Pseudidarnes* males are usually winged, but wingless males of *Pseudidarnes minerva* were recorded with very low frequencies (Cook et al. 1997, Early 2000). The small wingless males mate with females inside the figs even before females leave their galls, while winged males usually (but not always) disperse before mating (Cook et al. 1997).

In this paper we describe and illustrate six previously unknown species (two from Australia and four from Papua New Guinea). Redescriptions are also provided for *Pseudidarnes minerva* and *Pseudidarnes flavicollis*. We finally elaborated both dichotomous and interactive online keys to the known species of *Pseudidarnes*.

## Methods

### Specimen sampling and morphological study

Maturing fig syconia were collected, opened, and transferred to tissue bags until the wasps emerge, which happens after a few hours - days. Wasps were killed using acetate and transferred to 70% ethanol. Most geographical coordinates and altitudes were estimated using label information. Field recorded coordinates were provided when available. Field-collected specimens were dehydrated through an ethanol and HMDS series (Heraty and Hawks 1998) and then mounted on cards following Noyes (1982). Morphological terminology follows Gibson (1997). Material examined sections of species descriptions were prepared using AUTOMATEX (Brown 2013), and posteriorly refined.

Type and specimen depositories, and their respective curators are:

ASCI Australia, New South Wales, Orange, Orange Agricultural Institute, Agricultural Scientific Collections Unit (Peter Gillespie).

BMNH United Kingdom, London, The Natural History Museum [formerly British Museum (Natural History)](Natalie Dale-Skey Papilloud).

CBGP France, Montpellier. Centre de Biologie pour la Gestion des Populations (Emmanuelle Artige).

SAMC South Africa, Cape Town, Iziko South African Museum (Simon Van Noort).

### Illustration

To produce high quality images, some specimens were point-mounted on grey card in order to avoid loss of contrast caused by white background. Images were produced with an EntoVision Premium Portable Imaging System, comprising a Leica M16 zoom lens, a JVC KY-75U 3CCD digital camera and a portable computer workstation running EntoVision Imaging Suite software (GT Vision, Hagerstown, MD U.S.A.). Cartograph v5.6.0 (Microvision, Evry, France) software was subsequently used to merge an image series (representing about ten to twenty focal planes), producing a single image with increased depth of field. Illumination was achieved using a “quadrant” setup, with four fibre optic light guides stemming from two individual light sources (Leica CLS 150 X), similar to the one described by Buffington and Gates (2008). Images were edited using Adobe Photoshop CS4© software.

## Results

### Pseudidarnes

Girault, 1927

http://species-id.net/wiki/Pseudidarnes

http://www.figweb.org/Fig_wasps/Agaonidae/Sycophaginae/Pseudidarnes/index.htm

#### Type species.

*Pseudidarnes minerva* Girault, 1927, by monotypy.

#### Diagnosis.

*Female*. Body length (excluding ovipositor) 2.3–3.7 mm. Body colour variable, yellow to dark brown, sometimes with green metallic tinge.

*Head*. Face sculpture smooth to reticulate or slightly engraved, sometimes punctate. Antennae inserted well above to slightly below the middle line of compound eyes, but never very close to the clypeal margin. Toruli separated by one torulus diameter or less. Clypeal margin bilobed. Maxillary palpi composed of four segments. Labial palpi composed of three segments. Supraclypeal area delimited by subantennal grooves. Antenna with 13 segments (two anelli), and a 14^th^ segment very short and unconspicuous. Funicular segments 1–2 × as long as wide.

*Mesosoma*. Pronotum 1–2 × as long as high in lateral view. Notauli complete, deep and at least slightly crenulated. Mesoscutum as long as wide or longer than wide. Axilullar sulcus straight to slightly concave. Propodeum transverse, smooth to slightly reticulate and sometimes with a median sulcus. Wings hyaline, sometimes slightly infuscate medially. Marginal vein as long as stigmal vein, or longer. Postmarginal vein present (very short in *Pseudidarnes cooki* sp. n.). Marginal and postmarginal vein sometimes particularly widened (as Figs 4D, 6D, 8D, 10D & 16D).

*Metasoma*. First metasomal segment petiolate (petiole short in *Pseudidarnes acaudus* sp. n.). Ovipositor sheaths as long as body or shorter (very short in *Pseudidarnes acaudus* sp. n.).

*Male*. Very similar to female, usually slender and sometimes showing tinge variation. Male with very different colour patterns in *Pseudidarnes astridae* sp. n. (Figs 3–6).

#### Key to species of *Pseudidarnes* Girault (based mainly on females)

Online dichotomous and multi-access interactive LUCID keys to *Pseudidarnes* species are available at: http://www.figweb.org/Fig_wasps/Agaonidae/Keys/index.htm

**Table:** 

1	Ovipositor sheaths extremely short, only weakly protruding beyond metasoma apex (Figure 1A). Pedicel elongated, slightly shorter than the scape (Fig. 1D). Mesosoma entirely brown (Fig. 2A)	*Pseudidarnes acaudus* sp. n.
–	Ovipositor sheaths long, distinctly protruding beyond metasoma apex. Pedicel clearly shorter than the scape, at most 0.5 × the scape length. Mesosoma colour different, metallic or, when brown, with at least the pronotum yellow in lateral view	2
2	Pronotum yellow, without metallic tinge. Mesoscutum with irregular transverse striae (Fig. 4A, 8A, 16A). Marginal and postmarginal veins widened (Figs 4F, 8F, 16F).	3
–	Mesosoma metallic green, including pronotum. Mesoscutum smooth or reticulated. Marginal and postmarginal veins not widened	5
3	Head and mesosoma excluding pronotum with metallic tinge (Figs 15B, E, 16A). Propodeum with a well delimitated and carinulated median sulcus, extending to the posterior margin of the sclerite (Fig. 16B). Metascutellum crenulated (Fig. 16B)	*Pseudidarnes flavicollis* Bouček
–	Body without metallic tinge. Median sulcus of propodeum unconspicuous or absent. Metascutellum not crenulated	4
4	Mesosoma brown in dorsal view (Fig. 8A). Metascutellum with faint longitudinal striae (Figs 8B, 10B). Propodeum without median line (Figs 8B, 10B)	*Pseudidarnes badiogeminus* sp. n.
–	Mesosoma yellow in dorsal view (Fig. 4A, brown in males, but at least mesoscutellum yellow, Fig. 6A). Metascutellum and median area of propodeum with irregular transverse rugae (Figs 4B, 6B).	*Pseudidarnes astridae* sp. n.
5	Mesosoma entirely smooth and shiny (Figs 19B, 20A, 21B, 22A). First funicular segment 2 × as long as wide (Figs 19D, 21D). Distal antennomeres not forming a definite clava (Figs 19C, 21C). Propodeum with a very short median line (Figs 20B, 22B).	*Pseudidarnes laevis* sp. n.
–	Mesosoma sculpture mostly reticulate. First funicular segment ca. 1–1.5 × as long as wide. Distal antennomeres forming a definite clava. Propodeum medially with a deep carinulated sulcus, at least on the anterior half of the sclerite	6
6	Ovipositor sheaths short, about as long as the metasoma (Fig. 11A). Propodeum with a crenulated median sulcus extending to the posterior margin (Figs 12B, 14B)	*Pseudidarnes cooki* sp. n.
–	Ovipositor sheaths longer than metasoma. Propodeal median sulcus not reaching the posterior margin	7
7	Petiole transverse in dorsal view (Fig. 18D). Median sulcus of the propodeum broad and extending over the anterior half of the sclerite (Fig. 18B). Postmarginal vein shorter than the stigmal (Fig. 18F)	*Pseudidarnes kjellbergi* sp. n.
–	Petiole longer than wide in dorsal view (Figs 24D, 26D). Median sulcus of the propodeum extending over most of the propodeum length, not reaching the posterior margin (Figs 24B, 26B). Postmarginal veiwn as long as the stigmal (Figs 24F, 26F)	*Pseudidarnes minerva* Girault

### Species descriptions

#### Pseudidarnes acaudus

Farache & Rasplus sp. n.

http://zoobank.org/ECCB10FF-4783-45C9-A97E-02E1E27600CF

http://species-id.net/wiki/Pseudidarnes_acaudus

Figures 1 –2

##### Material examined.

*Holotype*. ♀, **PAPUA NEW GUINEA:** Crater Mountain, -6.58°, 145.08°, 2000m, V.1990, McKee A., ex *Ficus* sp. (CBGP).

*Paratype*. ♀, same data as holotype, (CBGP).

##### Diagnosis.

Pedicel elongated, slightly shorter than the scape. Mesosoma entirely brown. Petiole short, transverse in dorsal view. Ovipositor sheaths extremely short, only weakly protruding beyond metasoma apex.

##### Description.

*Female*. Body length 2.6 mm. Metallic tinge absent or very feeble. Predominantly brown. Scape and pedicel yellow brown. Head darker than mesosoma. Petiole yellow. Legs predominantly yellow, coxae almost concolorous with mesosoma. Remaining leg segments predominantly yellow and brown.

*Head*. Antennae inserted far above the middle line of compound eyes. Scape slightly longer than pedicel. Pedicel very elongated (more than 2 × as long as wide), slender, and longer than first funicular segment. Anelli almost as long as wide, proximal anellus longer than wide. First funicular segment approximately 1.5 × as long as wide. Distal antennomeres not forming a distinct clava. Face sculpture slightly engraved. Face pilosity short and sparse. Supraclypeal area narrow, its delimiting sulci converging near epistomal groove, and its sculpture barely rugose. Lateral ocelli nearly 1 × its diameter far from the eye margin.

*Mesosoma*. Pronotum longer than high in lateral view. Mesoscutum slightly engraved reticulate. Frenal sulcus smooth. Mesepimeron sculpture mostly smooth, slightly engraved. Metascutellum very short and smooth, inconspicuous, and almost completely covered by frenum. Propodeum with a well delimited and slightly carinulated median sulcus, which extends to the posterior margin of the sclerite. Propodeum sculpture smooth, slightly rugose. Wings hyaline, with sparse pilosity. Marginal and postmarginal vein not particularly widened. Postmarginal vein as long as stigmal vein.

*Metasoma*. Petiole smooth and transverse in lateral view. Petiole dorsally without a longitudinal median sulcus. Ovipositor sheaths extremely short, only weakly protruding beyond metasomal apex.

**Figure 1.**
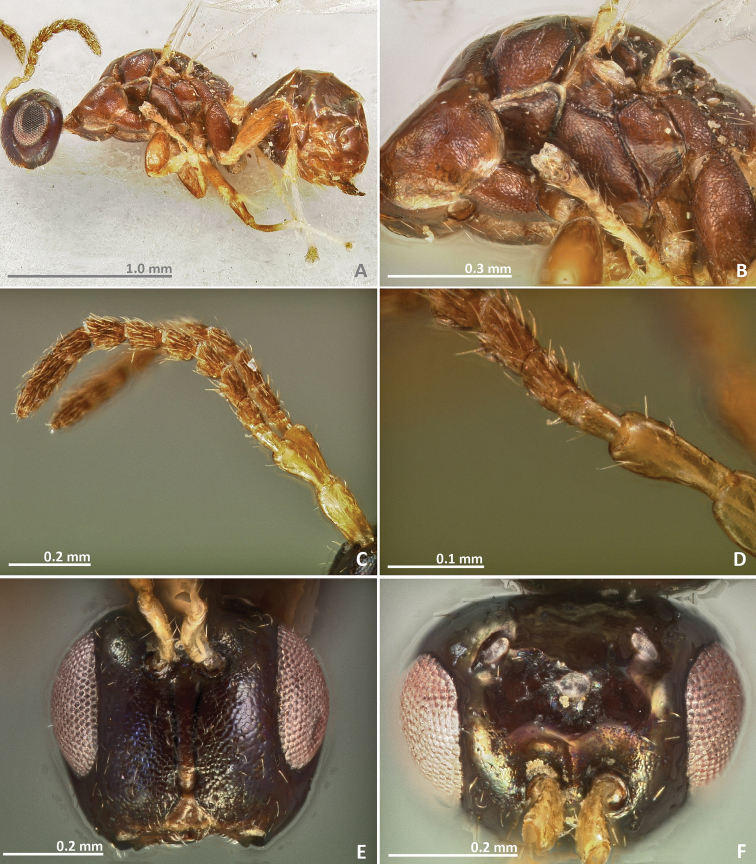
*Pseudidarnes acaudus* sp. n. female. **A** habitus lateral **B** mesosoma lateral **C** antenna **D** anelli **E** head, anterior view **F** vertex, dorsal view.

**Figure 2.**
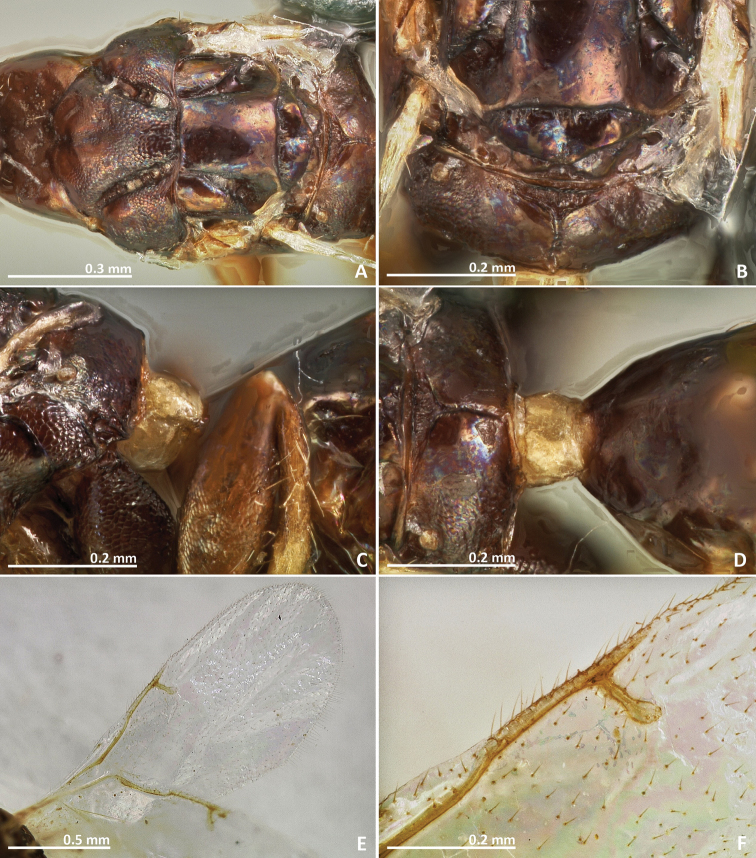
*Pseudidarnes acaudus* sp. n. female. **A** mesosoma dorsal **B** propodeum dorsal **C** petiole lateral view **D** petiole dorsal view **E** wing **F** detail of venation.

*Male*. Unknown.

##### Etymology.

The specific name refers to the short ovipositor sheaths exhibited by this species.

##### Biology.

Associated with an undetermined *Ficus* species collected in Papua New Guinea. Reared together with *Pseudidarnes laevis* sp. n., but less abundant than the later.

#### Pseudidarnes astridae

Farache & Rasplus sp. n.

http://zoobank.org/FBBF3AC7-E935-40E8-AF15-FA87970689EC

http://species-id.net/wiki/Pseudidarnes_astridae

**Figure 3.**
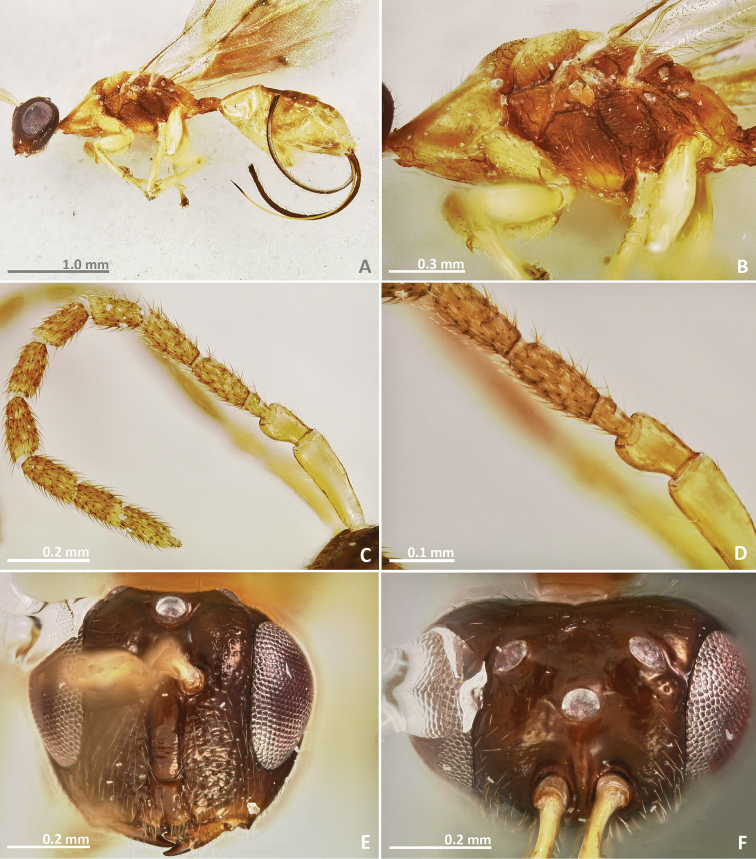
*Pseudidarnes astridae* sp. n. female. **A** habitus lateral **B** mesosoma lateral **C** antenna **D** anelli **E** head, anterior view **F** vertex, dorsal view.

Figures 3 [Fig F4] [Fig F5] –6

##### Material examined.

*Holotype*. ♀, **PAPUA NEW GUINEA: East New Britain:** Raunsepna, North Baining Mountains, -4.433°, 151.783°, 1000m, 26.II.1999, Vaamonde CL, ex *Ficus xylosycia* CLV11 (CBGP).

*Paratype*. ♂ Same data as holotype (CBGP).

##### Diagnosis.

Body without metallic tinge. Pronotum long, nearly 1.5–2 × as long as high in lateral view. Mesoscutum with faint irregular transverse striae. Median area of metascutellum and median area of propodeum with irregular transverse rugae. Marginal and postmarginal vein widened.

##### Description.

*Female*. Body length 3.4 mm. Ovipositor sheaths length 1.8 mm. Metallic tinge absent. Predominantly yellow. Head dark brown. Mesepisternum, mesepimeron, and metapleuron predominantly brown.

*Head*. Antennae inserted far above the middle line of compound eyes. Scape more than 2 × as long as pedicel. Pedicel elongated, slender, but shorter than first funicular segment. Anelli almost as long as wide. First funicular segment 2 × as long as wide. Distal antennomeres not forming a distinct clava. Face sculpture smooth with sparse punctures, lower face slightly engraved. Face pilosity long and dense. Supraclypeal area wide, its delimiting sulci not converging near epistomal groove, and its sculpture mostly smooth. Lateral ocelli 0.5 × its own diameter far from the eye margin.

*Mesosoma*. Pronotum long, nearly 1.5–2 × as long as high in lateral view. Mesoscutum with faint transversal striae. Mesoscutellum smooth. Frenal sulcus sparsely crenulated. Mesepimeron sculpture mostly smooth, slightly striate. Metascutellum longer than frenum and smooth, with faint and irregular transverse rugae at the median line. Propodeum sculpture mostly smooth. Median line of propodeum with irregular transverse rugae. Wings with rather dense pilosity, and medially infuscate. Marginal and postmarginal vein widened. Postmarginal vein shorter than stigmal vein.

*Metasoma*. Petiole rugose, 1.7 × as long as high in lateral view. Petiole dorsally without a longitudinal median sulcus. Ovipositor sheaths long, distinctly protruding beyond metasoma apex. Ovipositor sheaths length 2.8 × hind tibia length and 0.5 × body length.

**Figure 4. F4:**
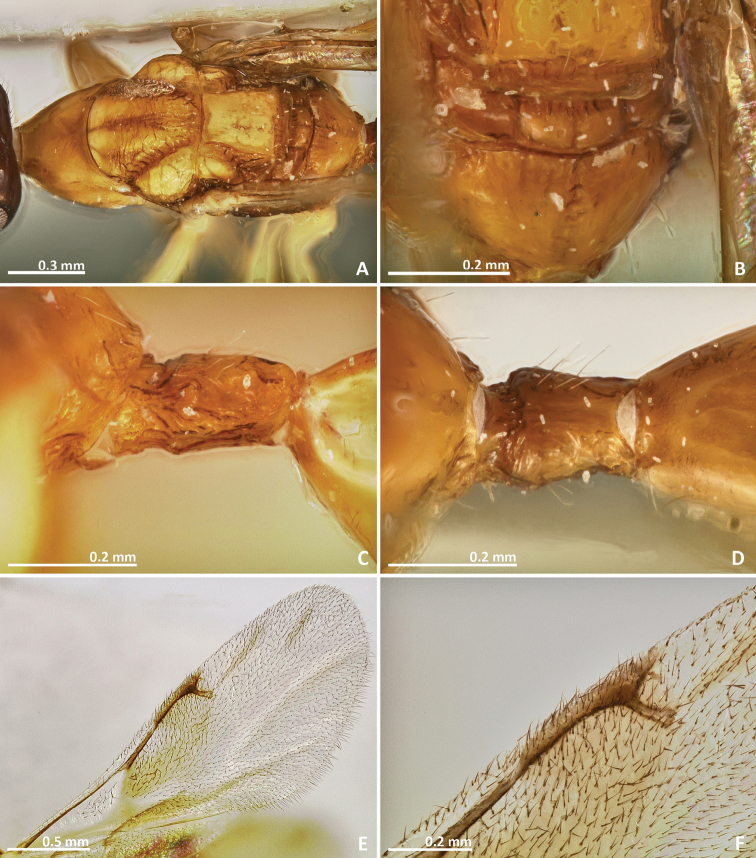
*Pseudidarnes astridae* sp. n. female. **A** mesosoma dorsal **B** propodeum dorsal **C** petiole lateral view **D** petiole dorsal view **E** wing **F** detail of venation.

*Male*. Body length 4.2 mm. Characters agreeing with females, except the following. Body colour browner, mesoscutellum yellow in dorsal view. Posterior ocelli contiguous to the eye margin, and larger. Wing infuscation more pronounced.

**Figure 5. F5:**
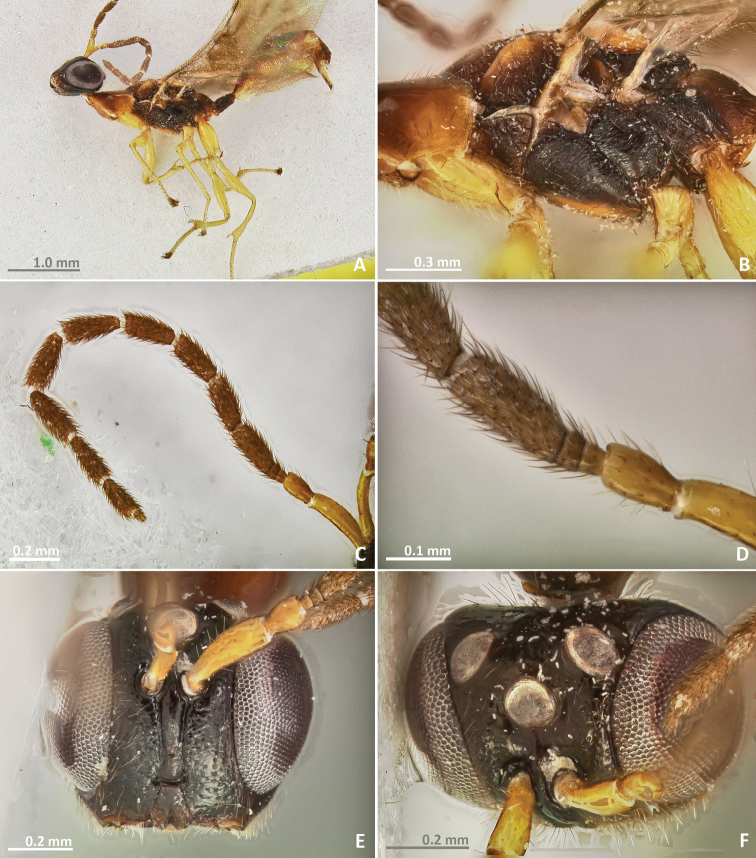
*Pseudidarnes astridae* sp. n. male. **A** habitus lateral **B** mesosoma lateral **C** antenna **D** anelli **E** head, anterior view **F** vertex, dorsal view.

**Figure 6.**
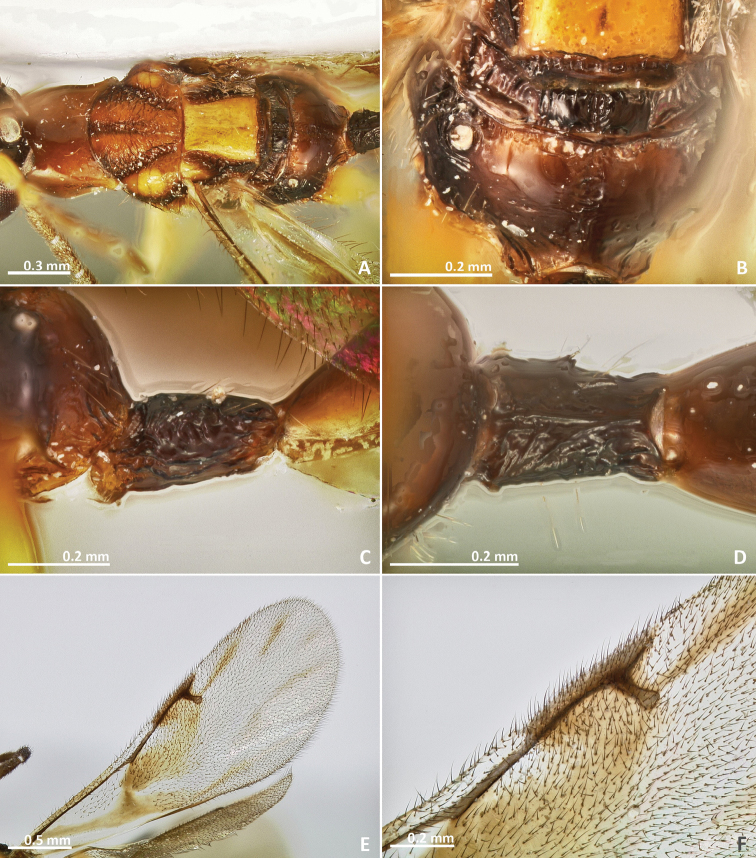
*Pseudidarnes astridae* sp. n. male. **A** mesosoma dorsal **B** propodeum dorsal **C** petiole lateral view **D** petiole dorsal view **E** wing **F** detail of venation.

##### Etymology.

The specific name is dedicated to our friend Astrid Cruaud for the long long walks we share together in the jungles of the world, trying to find fig trees.

##### Biology.

Reared from syconia of *Ficus xylosycia* Diels. *Ficus xylosycia* hosted three *Pseudidarnes* species. *Pseudidarnes badiogeminus* sp. n. was collected together with *Pseudidarnes astridae* sp. n. in New Britain, whereas *Pseudidarnes flavicollis* Bouček was collected in Bulolo. We are convinced that the host identification is correct for both samplings and that the guilds of non-pollinating fig wasps may vary with geography.

#### Pseudidarnes badiogeminus

Farache & Rasplus sp. n.

http://zoobank.org/0EA058A5-E760-4114-8CBD-72DF1D488803

http://species-id.net/wiki/Pseudidarnes_badiogeminus

**Figure 7.**
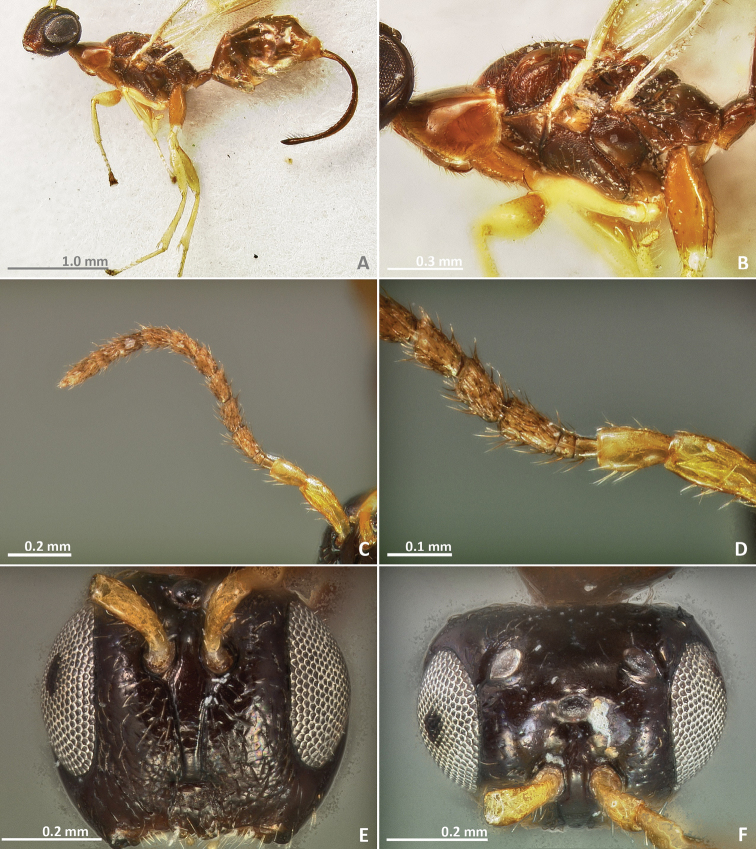
*Pseudidarnes badiogeminus* sp. n. female. **A** habitus lateral **B** mesosoma lateral **C** antenna **D** anelli **E** head, anterior view **F** vertex, dorsal view.

Figures 7 [Fig F8] [Fig F9] –10

##### Material examined.

*Holotype*. ♀, **PAPUA NEW GUINEA: East New Britain:** Raunsepna, North Baining Mountains, -4.433°, 151.783°, 1000m, 26.II.1999, Vaamonde CL, ex *Ficus xylosycia* CLV11 (CBGP).

*Paratype*. 1♀ 1♂ same data as holotype (CBGP).

##### Diagnosis.

Pronotum long, nearly 1.5–2 × as long as high in lateral view. Mesosoma brown in dorsal view. Mesoscutum with irregular transverse rugae. Metascutellum with faint longitudinal striae. Marginal and postmarginal veins widened. Median line of propodeum absent

##### Description.

*Female*. Body length 3 mm. Ovipositor sheaths length 1.6 mm. Metallic tinge absent. Body colour predominantly brown. Scape and pedicel yellow. Flagellomeres yellow brown. Head dark brown. Pronotum yellow brown laterally. Legs yellow, coxae browner.

*Head*. Antennae inserted far above the middle line of compound eyes. Scape nearly 2 × as long as pedicel. Pedicel elongated, slender, and as long as first funicular segment. Proximal anellus longer than wide. First funicular segment 2 × as long as wide. Distal antennomeres not forming a distinct clava. Face sculpture engraved, slightly rugose. Face pilosity short and sparse. Supraclypeal area narrow, its delimiting sulci converging near epistomal groove, and its sculpture barely rugose. Lateral ocelli 0.5 × its own diameter far from the eye margin.

*Mesosoma*. Pronotum long, nearly 1.5–2 × as long as high in lateral view. Mesoscutum transversally striate. Mesoscutellum mostly smooth. Frenal sulcus with shallow crenulation. Mesepimeron sculpture mostly smooth, slightly striate. Metascutellum longer than frenum, with faint longitudinal striae. Propodeum smooth, without median line. Wings with rather dense pilosity, and medially infuscate. Marginal and postmarginal vein widened. Postmarginal vein longer than stigmal vein.

*Metasoma*. Petiole slightly rugose, 1.7 × as long as high in lateral view. Petiole dorsally without a longitudinal median sulcus. Ovipositor sheaths long, distinctly protruding beyond metasoma apex. Ovipositor sheaths length 2.7 × hind tibia length and 0.5 × body length.

**Figure 8. F8:**
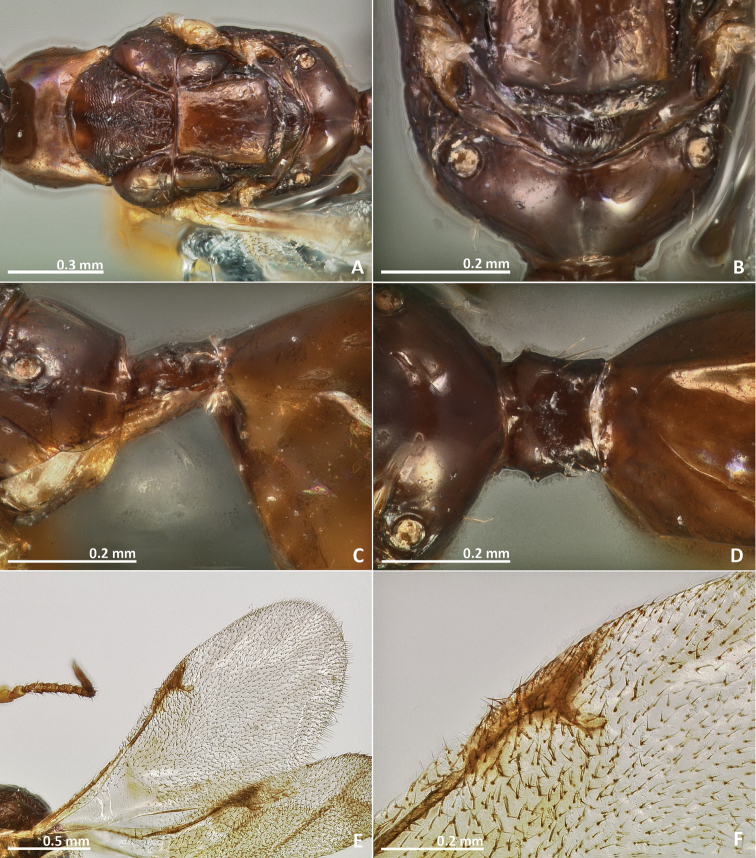
*Pseudidarnes badiogeminus* sp. n. female. **A** mesosoma dorsal **B** propodeum dorsal **C** petiole lateral view **D** petiole dorsal view **E** wing **F** detail of venation.

*Male*. Body length 3.2 mm. Characters agreeing with females, except the following: ocelli larger and contiguous to the eye margin. Pedicel slightly shorter.

**Figure 9. F9:**
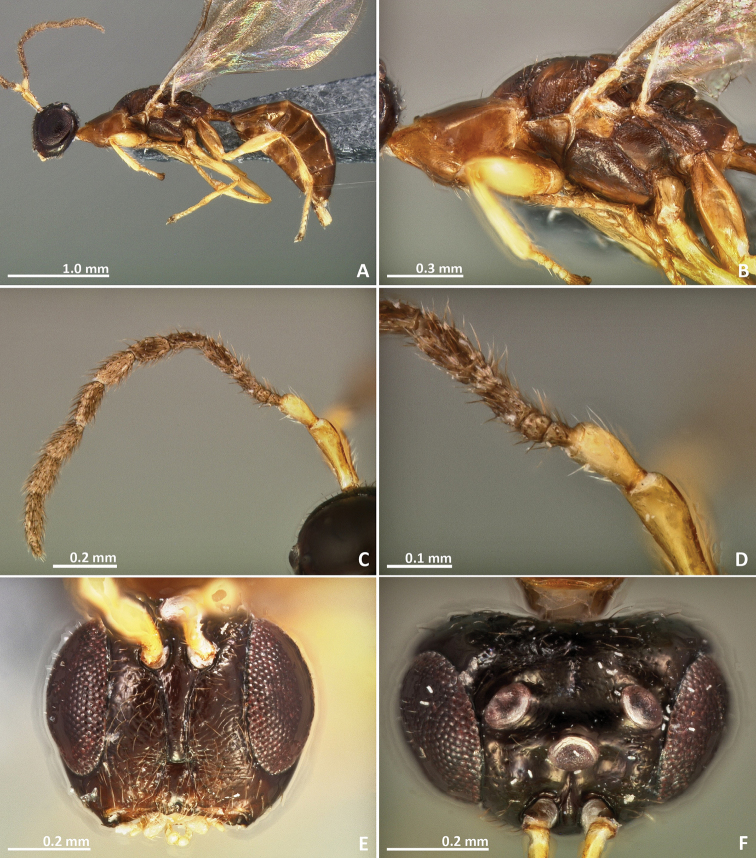
*Pseudidarnes badiogeminus* sp. n. male. **A** habitus lateral **B** mesosoma lateral **C** antenna **D** anelli **E** head, anterior view **F** vertex, dorsal view.

**Figure 10.**
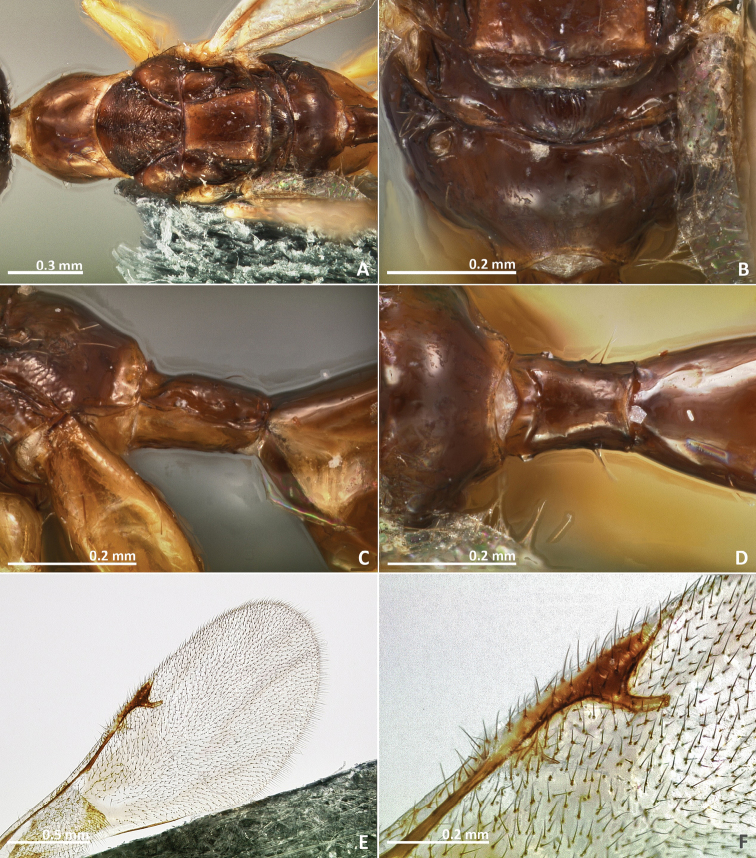
*Pseudidarnes badiogeminus* sp. n. male. **A** mesosoma dorsal **B** propodeum dorsal **C** petiole lateral view **D** petiole dorsal view **E** wing **F** detail of venation.

##### Etymology.

The specific name refers to the similarity of *Pseudidarnes badiogeminus* with *Pseudidarnes astridae*, but showing different colour.

##### Biology.

Collected from syconia of *Ficus xylosycia*. See *Pseudidarnes astridae* for further information.

#### Pseudidarnes cooki

Farache & Rasplus sp. n.

http://zoobank.org/2C54B54B-5925-45BD-A1CA-1440704749C8

http://species-id.net/wiki/Pseudidarnes_cooki

**Figure 11.**
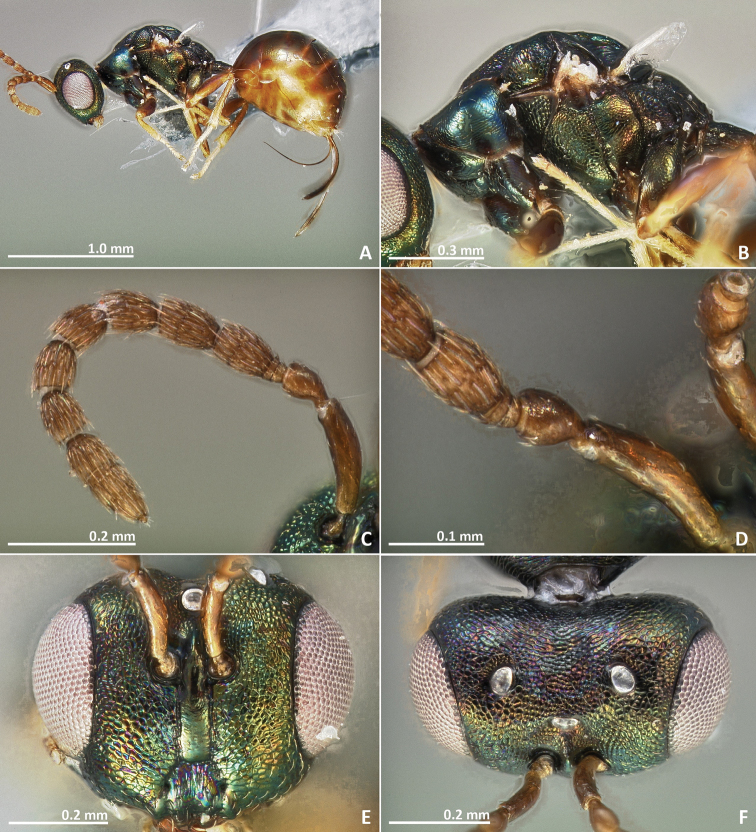
*Pseudidarnes cooki* sp. n. female. **A** habitus lateral **B** mesosoma lateral **C** antenna **D** anelli **E** head, anterior view **F** vertex, dorsal view.

Figures 11 [Fig F12] [Fig F13] –14

Pseudidarnes sp. ex *Ficus obliqua*; Cruaud et al. (2011a) BMC Evolutionary Biology, 11: 15pp. [phylogenetic position]
Pseudidarnes sp. ex *Ficus obliqua*; Cruaud et al. (2011b) Journal of Biogeography, 38: 209–225. [biogeography]
Pseudidarnes sp.; Segar ST, Cook JM (2012) Ecological Entomology, 37(5), 342–349. [ecology]

##### Material examined.

*Holotype*. ♀, **AUSTRALIA: Queensland:** Cairns, Rex Lookout, -16.65°, 145.56°, 100m, 13.I.1999, Rasplus J.Y., ex *Ficus obliqua* (CBGP).

*Paratypes*. **AUSTRALIA: Queensland:** Cairns, Rex Lookout, -16.65°, 145.56°, 100m, 7♀, 1♂, 13.I.1999, Rasplus J.Y., ex. *Ficus obliqua* (CBGP), North of Cairns, Costal road, -16.65°, 145.56°, 100m, 1♀, 27.X.2005, Jousselin E. & Coeur d’Acier A., ex *Ficus obliqua*, n° JRAS01422 (CBGP), Port Douglas, -16.483230°, 145.464058°, 10m, 3♀, 28.X.2005, Jousselin E. & Coeur d’Acier A., ex *Ficus obliqua*, n° JRAS01429 (1 ♀ CBGP, 1 ♀ BMNH, 1 ♀ SAMC).

##### Diagnosis.

Metallic tinge present at least in some body regions. Mesosoma sculpture mostly reticulate. Propodeum with a crenulated median sulcus extending to the posterior margin. Postmarginal vein shorter than stigmal vein. Ovipositor sheaths short, about as long as the metasoma.

##### Description.

*Female*. Body length 2.3 mm. Ovipositor sheaths length 0.9 mm. Metallic tinge present at least in some body regions. Predominantly dark green. Antennae brown. Coxae almost concolorous with mesosoma. Femora brown. Tibiae and tarsi predominantly yellow. Metatibia proximally yellow brown. Metasoma predominantly brown.

*Head*. Antennae inserted just above the middle line of compound eyes. Scape nearly 3 × as long as pedicel. Pedicel almost as long as wide, pyriform, and shorter than first funicular segment. Anelli transverse. First funicular segment approximately 1.5 × as long as wide. Distal antennomeres forming a distinctive clava. Face sculpture reticulate. Face pilosity short and sparse, becoming longer near oral margin and eyes. Supraclypeal area wide, its delimiting sulci not converging near epistomal groove, and its sculpture mostly smooth. Lateral ocelli 1 × its own diameter far from the eye margin.

*Mesosoma*. Pronotum short, nearly as long as high in lateral view. Mesoscutum strongly reticulate. Mesoscutellum reticulate. Frenal sulcus densely crenulated. Mesepimeron sculpture reticulate. Metascutellum longer than frenum, reticulate. Propodeum with a well delimited and carinulated median sulcus, extending to the posterior margin of the sclerite. Propodeum sculpture reticulate, smooth near the proximal region of median line of propodeum. Wings hyaline, with sparse pilosity. Marginal and postmarginal vein not particularly widened. Postmarginal vein shorter than stigmal vein.

*Metasoma*. Petiole rugose, 1.5 × as long as high in lateral view. Petiole dorsally with a longitudinal median sulcus. Ovipositor sheaths long, distinctly protruding beyond metasoma apex. Ovipositor sheaths length 2.25 × hind tibia length, 0.4 × body length.

**Figure 12. F12:**
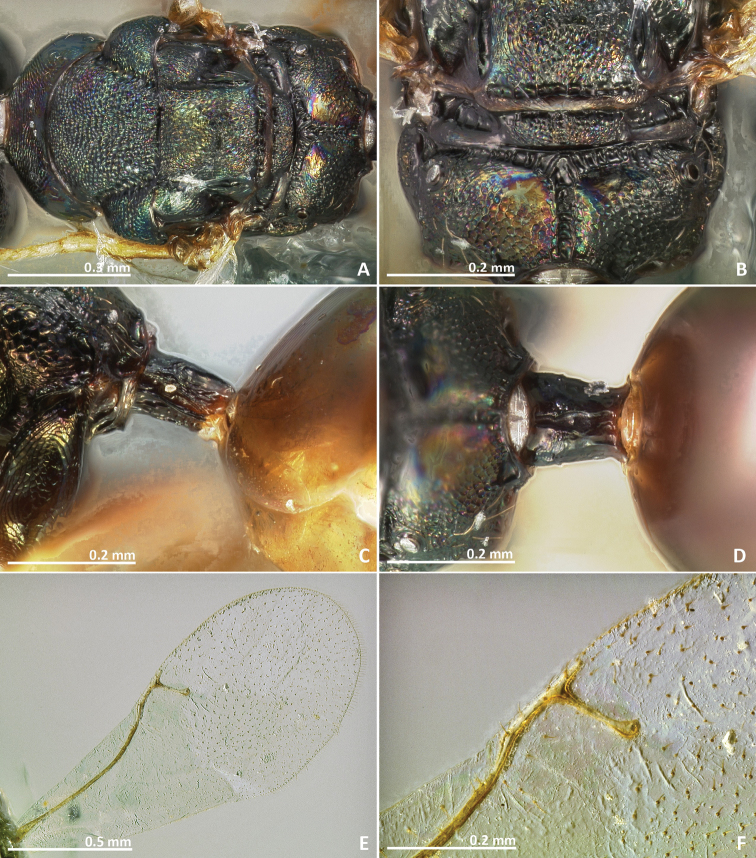
*Pseudidarnes cooki* sp. n. female. **A** mesosoma dorsal **B** propodeum dorsal **C** petiole lateral view **D** petiole dorsal view **E** wing **F** detail of venation.

*Male*. Body length 2.4 mm. Characters agreeing with the females, except the following. Body slender. Antenna more yellow and inserted at the middle line of compound eyes or slightly below. Ocelli larger. Body sculpture fainter. Petiole more brown. Wings more pilose.

**Figure 13. F13:**
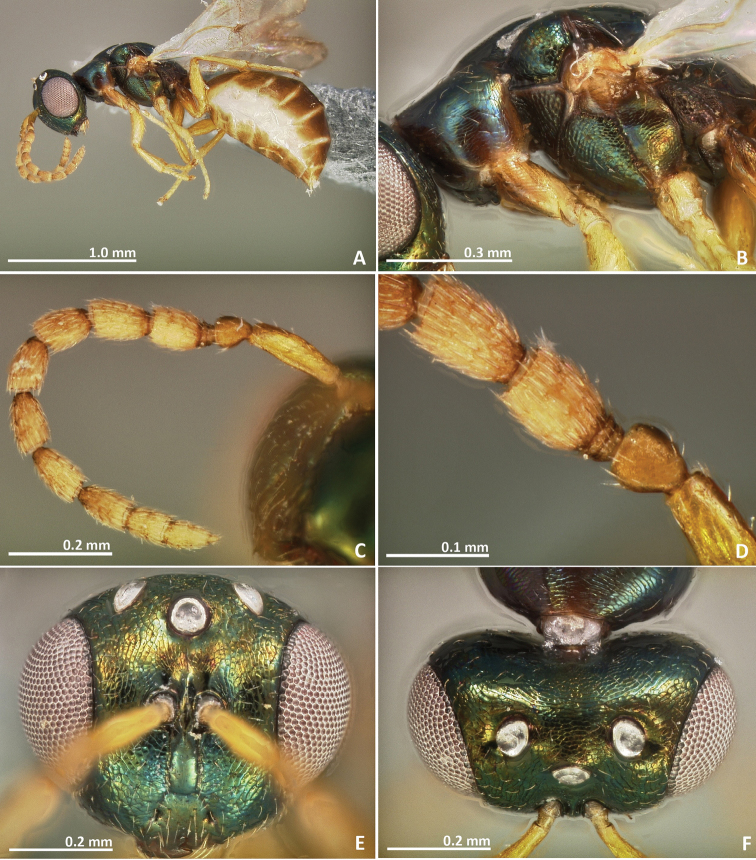
*Pseudidarnes cooki* sp. n. male. **A** habitus lateral **B** mesosoma lateral **C** antenna **D** anelli **E** head, anterior view **F** vertex, dorsal view.

**Figure 14.**
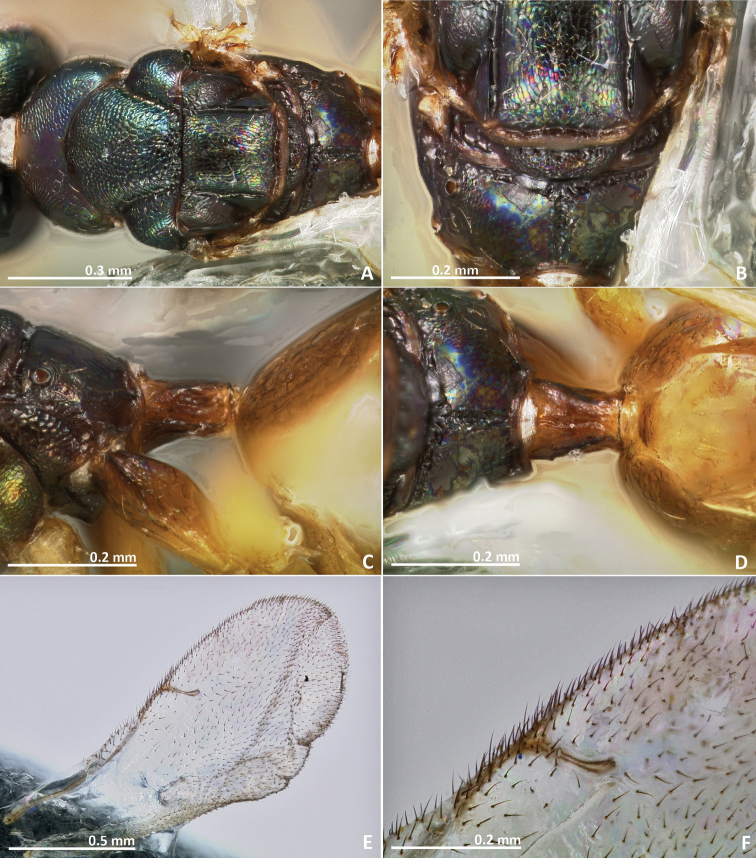
*Pseudidarnes cooki* sp. n. male. **A** mesosoma dorsal **B** propodeum dorsal **C** petiole lateral view **D** petiole dorsal view **E** wing **F** detail of venation.

##### Etymology.

The specific name is dedicated to our friend and colleague Dr. James Cook, in recognition of his amazing contribution to our knowledge of fig wasps.

##### Biology.

This species is strictly associated with *Ficus obliqua* G. Forst. and was studied by Segar and Cook (2012) (referred as *Pseudidarnes* sp.). It was reared in low abundance (0.1 ± 0.05, mean ± *SE*) and there were rarely more than four wasps in the same syconium.

##### Molecular data.

GenBank sequences: COI HM770642; Cytb HM770596; EF-1a HM770545; rRNA 28S HM770704 (Cruaud et al. 2011a; Cruaud et al. 2011b).

#### Pseudidarnes flavicollis

Bouček, 1988

http://species-id.net/wiki/Pseudidarnes_flavicollis

Figures 15 –16

##### Material examined.

*Holotype*. ♀, **PAPUA NEW GUINEA: Bulolo:** Manki area, -5.37°, 144.18°, 700m, 22.VII.1981, Roberts H., ex *Ficus xylosycia* (BMNH) [examined].

*Paratype*. ♀, same data as holotype (BMNH) [examined].

##### Diagnosis.

Head and mesosoma excluding pronotum with metallic tinge. Pronotum long, nearly 1.5–2 × as long as high in lateral view. Mesoscutum with irregular transverse rugae. Marginal and postmarginal veins widened. Propodeum with a well delimitated and carinulated median sulcus, extending to the posterior margin of the sclerite. Metascutellum as well as lateral panel of metanotum crenulated.

##### Description.

*Female*. Body length 3.3 mm. Ovipositor sheaths length 1.6 mm. Metallic tinge present at least in some body regions. Predominantly yellow and green. Scape and pedicel yellow. Flagellomeres brown. Head green, green red near vertex. Pronotum yellow. Prepectus yellow brown. Remaining mesosoma geen to dark green, propodeum green brown. Legs predominantly yellow, metacoxa proximally brown. Petiole dark brown. Metasoma predominantly yellow, dorsally brown.

*Head*. Antennae inserted far above the middle line of compound eyes. Scape nearly 2 × as long as pedicel. Pedicel elongated, slender, and shorter than first funicular segment. Anelli transverse. First funicular segment 2 × as long as wide. Distal antennomeres not forming a distinct clava. Face sculpture smooth with sparse punctures, lower face with engraved transverse striae. Face pilosity short and sparse, becoming longer near oral margin and eyes. Supraclypeal area wide, its delimiting sulci not converging near epistomal groove, and its sculpture mostly smooth. Lateral ocelli contiguous to the eye margin.

*Mesosoma*. Pronotum long, nearly 1.5–2 × as long as high in lateral view. Mesoscutum transversally striate. Mesoscutellum smooth. Frenal sulcus densely crenulated. Mesepimeron sculpture slightly striate. Metascutellum as well as lateral panel of metanotum crenulated, metascutellum longer than frenum. Propodeum with a well delimited and carinulated median sulcus, extending to the posterior margin of the sclerite. Propodeum sculpture mostly smooth, slightly rugose laterally. Wings hyaline, with rather dense pilosity. Marginal and postmarginal vein widened. Postmarginal vein longer than stigmal vein.

*Metasoma*. Petiole rugose, 2 × as long as high in lateral view. Petiole dorsally without a longitudinal median sulcus. Ovipositor sheaths long, distinctly protruding beyond metasoma apex. Ovipositor sheaths length 2.7 × hind tibia length, 0.5 × body length.

**Figure 15.**
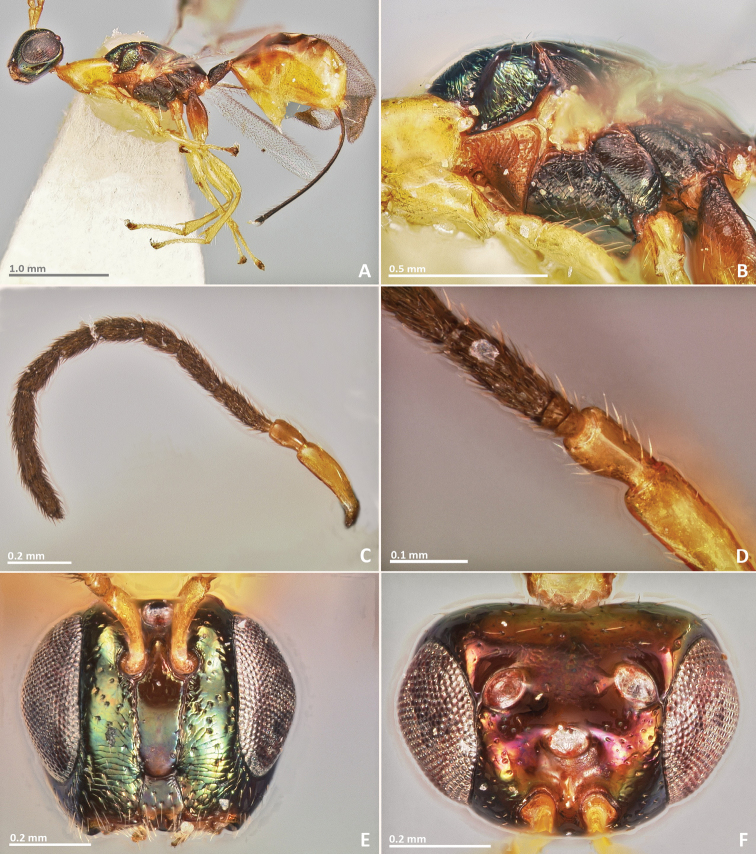
*Pseudidarnes flavicollis* Bouček, 1988, paratype, female. **A** habitus lateral **B** mesosoma lateral **C** antenna **D** anelli **E** head, anterior view **F** vertex, dorsal view.

**Figure 16.**
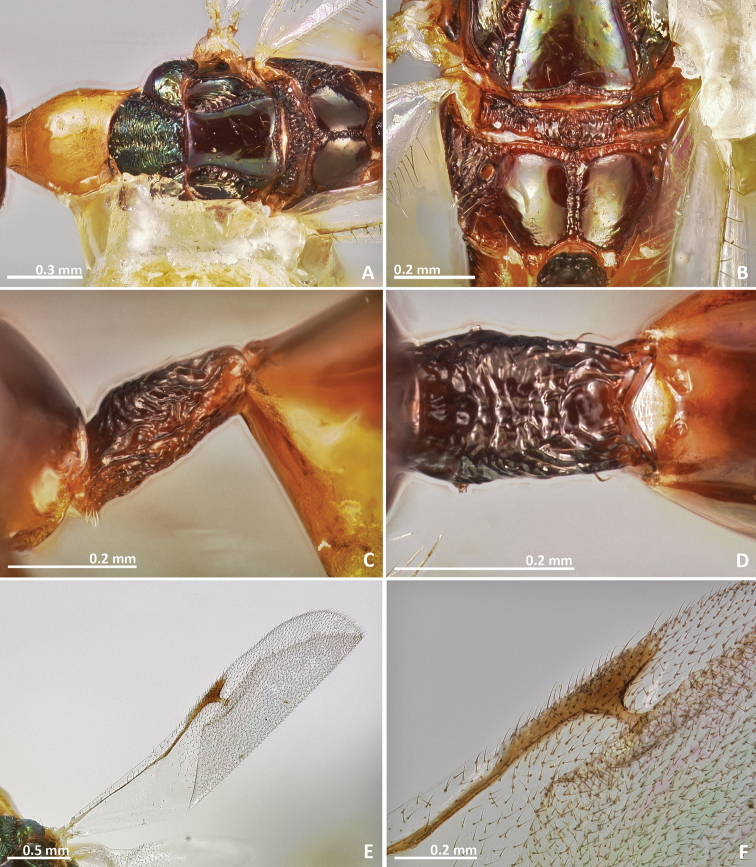
*Pseudidarnes flavicollis* Bouček, 1988, paratype, female. **A** mesosoma dorsal **B** propodeum dorsal **C** petiole lateral view **D** petiole dorsal view **E** wing **F** detail of venation.

*Male*. Unknown.

##### Biology.

Collected from syconia of *Ficus xylosycia* Diels. See *Pseudidarnes astridae* biology section.

#### Pseudidarnes kjellbergi

Farache & Rasplus sp. n.

http://zoobank.org/1DF43475-A3A3-431B-8B59-C3810F83970F

http://species-id.net/wiki/Pseudidarnes_kjellbergi

Figures 17 –18

##### Material examined.

*Holotype*. ♀, **AUSTRALIA: Kununarra:**, -15.8319°, 128.8564°, 80m, 20.X.1997, Dixon, D., ex *Ficus platypoda*, n° PhD 455 (CBGP).

*Paratype*. ♀, same data as holotype (CBGP).

##### Diagnosis.

Mesosoma metallic green. Mesosoma sculpture mostly reticulate. Median sulcus of the propodeum extending over the anterior half of the sclerite. Postmarginal vein shorter than the stigmal. Petiole transverse in dorsal view. Ovipositor sheaths longer than metasoma.

##### Description.

*Female*. Body length 3.1 mm. Ovipositor sheaths length 2.4 mm. Metallic tinge present at least in some body regions. Predominantly green. Antenna yellow brown. Coxae brown. Femora and tibiae predominantly brown. Tarsi yellow. Petiole brown. Metasoma green brown.

*Head*. Antennae inserted at the middle line of compound eyes. Scape nearly 3 × as long as pedicel. Pedicel almost as long as wide, pyriform, and as long as first funicular segment. Anelli transverse. First funicular segment approximately as long as wide. Distal antennomeres forming a distinctive clava. Face sculpture reticulate. Face pilosity short and sparse. Supraclypeal area wide, its delimiting sulci not converging near epistomal groove, and its sculpture mostly smooth. Lateral ocelli nearly 1 × its diameter far from the eye margin.

*Mesosoma*. Pronotum short, nearly as long as high in lateral view. Mesoscutum strongly reticulate. Mesoscutellum reticulate. Frenal sulcus densely crenulated. Mesepimeron sculpture slightly reticulate ventrally, becoming smooth in its medial and upper region. Metascutellum as long as frenum, reticulate. Propodeum with a broad crenulated median line extending over the anterior half of the sclerite. Median line very faint or absent in the posterior half of the propodeum. Propodeum sculpture engraved reticulate. Wings hyaline, with sparse pilosity. Marginal and postmarginal vein not particularly widened. Postmarginal vein shorter than stigmal vein.

*Metasoma*. Petiole slightly rugose, and transverse in lateral view. Petiole dorsally without a longitudinal median sulcus Ovipositor sheaths long, distinctly protruding beyond metasomal apex. Ovipositor sheaths length 3.8 × hind tibia length, 0.7 × body length.

**Figure 17.**
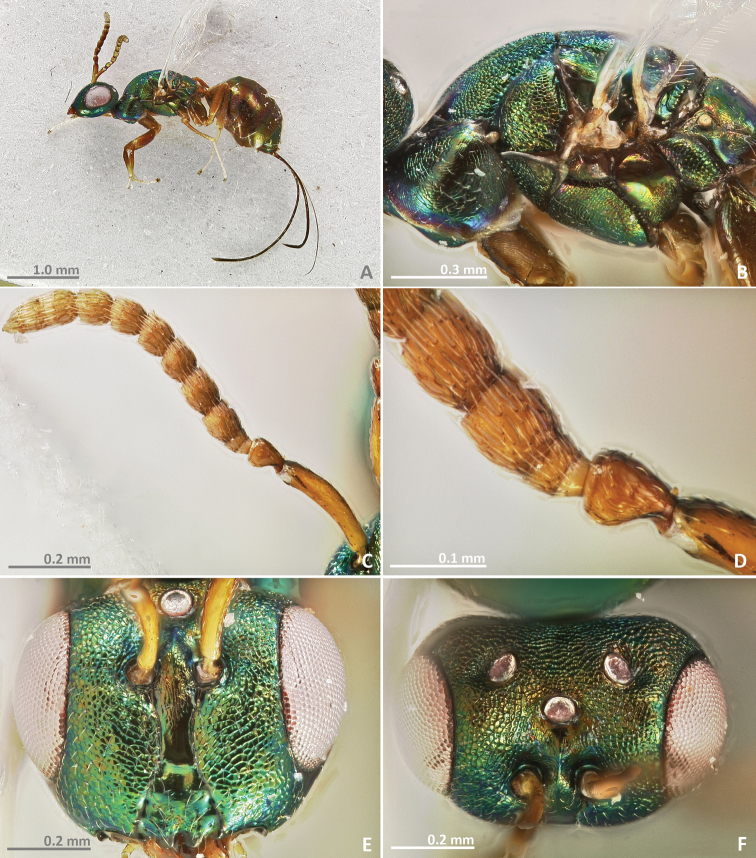
*Pseudidarnes kjellbergi* sp. n. female. **A** habitus lateral **B** mesosoma lateral **C** antenna **D** anelli **E** head, anterior view **F** vertex, dorsal view.

**Figure 18.**
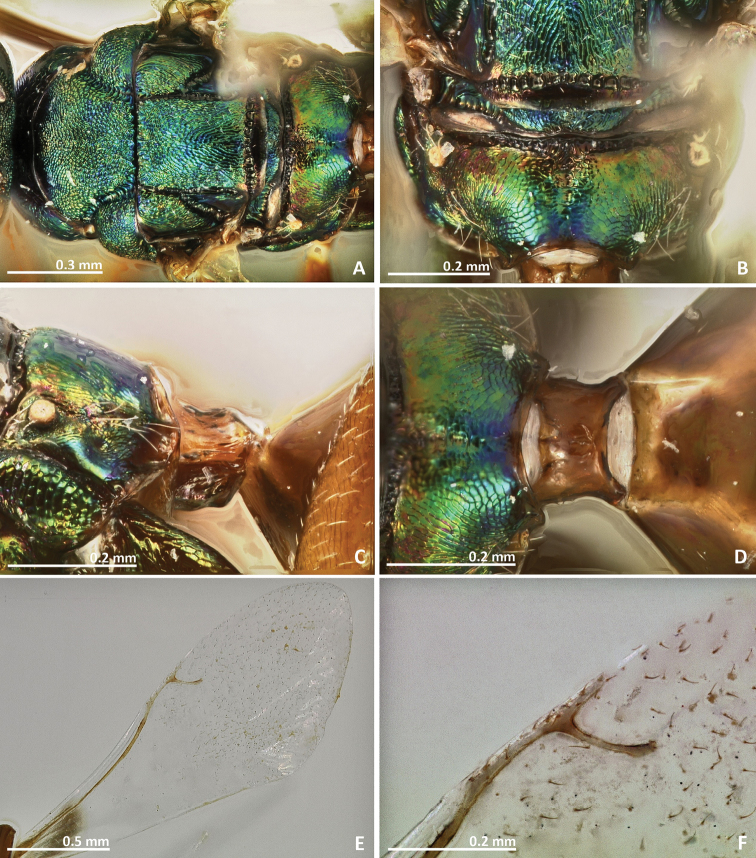
*Pseudidarnes kjellbergi* sp. n. female. **A** mesosoma dorsal **B** propodeum dorsal **C** petiole lateral view **D** petiole dorsal view **E** wing **F** detail of venation.

*Male*. Unknown.

##### Etymology.

The specific name is dedicated to our friend and colleague Dr. Finn Kjellberg, in recognition of his excellent work in fig wasps.

##### Biology.

Reared from syconia of *Ficus platypoda* (Miq.) A. Cunn. ex Miq.

#### Pseudidarnes laevis

Farache & Rasplus sp. n.

http://zoobank.org/475EA63C-EDE3-4BE9-819E-616FBEE94879

http://species-id.net/wiki/Pseudidarnes_laevis

**Figure 19.**
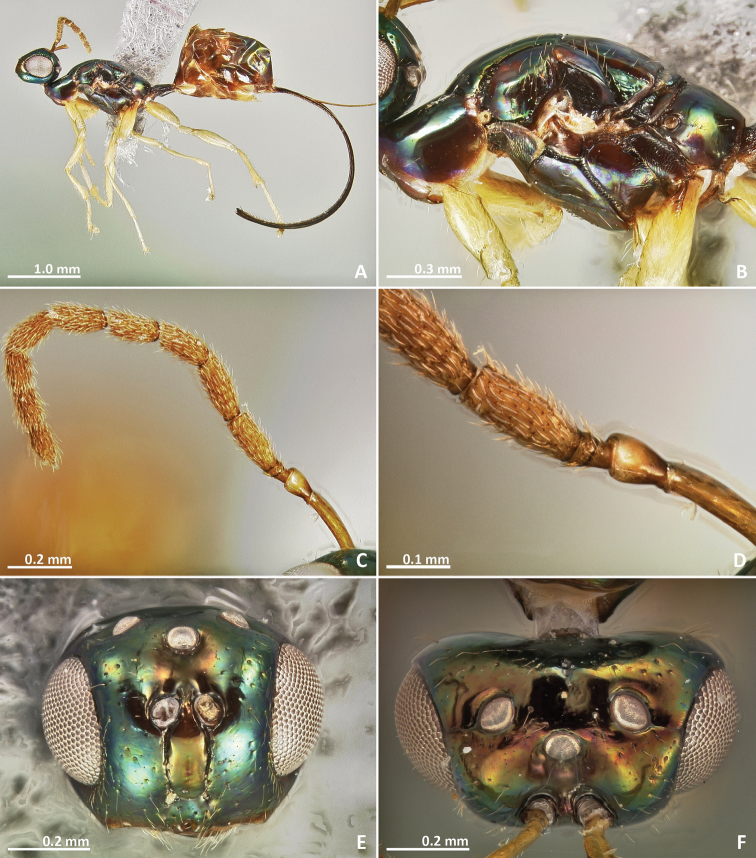
*Pseudidarnes laevis* sp. n. female. **A** habitus lateral **B** mesosoma lateral **C** antenna **D** anelli **E** head, anterior view **F** vertex, dorsal view.

Figures 19 [Fig F20] [Fig F21] –22

##### Material examined.

*Holotype*. ♀, **PAPUA NEW GUINEA:** Crater Mountain -6.58°, 145.08°, 2000m, V.1990, McKee A., ex *Ficus* sp., n° AM 451 (CBGP).

*Paratypes*. **PAPUA NEW GUINEA:** Crater Mountain -6.58°, 145.08°, 2000m, 20♀, 20♂, V.1990, McKee A., ex *Ficus* sp., n° AM 451 (17♀, 18♂ CBGP, 1♀, 1♂ BMNH, 1♀, 1♂ SAMC, 1♀ RPSP), 6♀, 1♂, V.1990, McKee A., ex *Ficus* sp., n° AM 550 (CBGP).

##### Diagnosis.

Mesosoma metallic green, entirely smooth and shiny. First funicular segment 2 × as long as wide. Distal antennomeres not forming a definite clava.

##### Description.

*Female*. Body length 3.7 mm. Ovipositor sheaths length 3.9 mm. Metallic tinge present at least in some body regions. Predominantly dark green. Antennae yellow brown. Legs yellow. Petiole dark brown. Metasoma predominantly brown, slightly green.

*Head*. Antennae inserted at the middle line of compound eyes or slightly above. Scape nearly 3 × as long as pedicel. Pedicel almost as long as wide, shorter than first funicular segment. Anelli transverse. First funicular segment 2 × as long as wide. Distal antennomeres not forming a distinct clava. Face sculpture smooth, with very sparse punctures. Face pilosity short and sparse. Supraclypeal area wide, its delimiting sulci not converging near epistomal groove, and its sculpture mostly smooth. Lateral ocelli nearly 1 × its diameter far from the eye margin.

*Mesosoma*. Pronotum longer than high in lateral view. Mesoscutum mostly smooth. Mesoscutellum smooth. Frenal sulcus densely crenulated. Mesepimeron sculpture mostly smooth. Metascutellum smooth, very short, inconspicuous, and almost completely covered by frenum. Propodeum with a vestigial median line not extending from the beginning of the proximal region. Propodeum sculpture smooth. Wings hyaline, with rather dense pilosity. Marginal and postmarginal vein not particularly widened. Postmarginal vein as long as stigmal vein, or slightly longer.

*Metasoma*. Petiole 2 × as long as high in lateral view. Petiole sculpture in lateral view slightly rugose. Petiole dorsally with a longitudinal median sulcus. Ovipositor sheaths long, distinctly protruding beyond metasoma apex. Ovipositor sheaths length 4.3 × hind tibia length, as long as body.

**Figure 20. F20:**
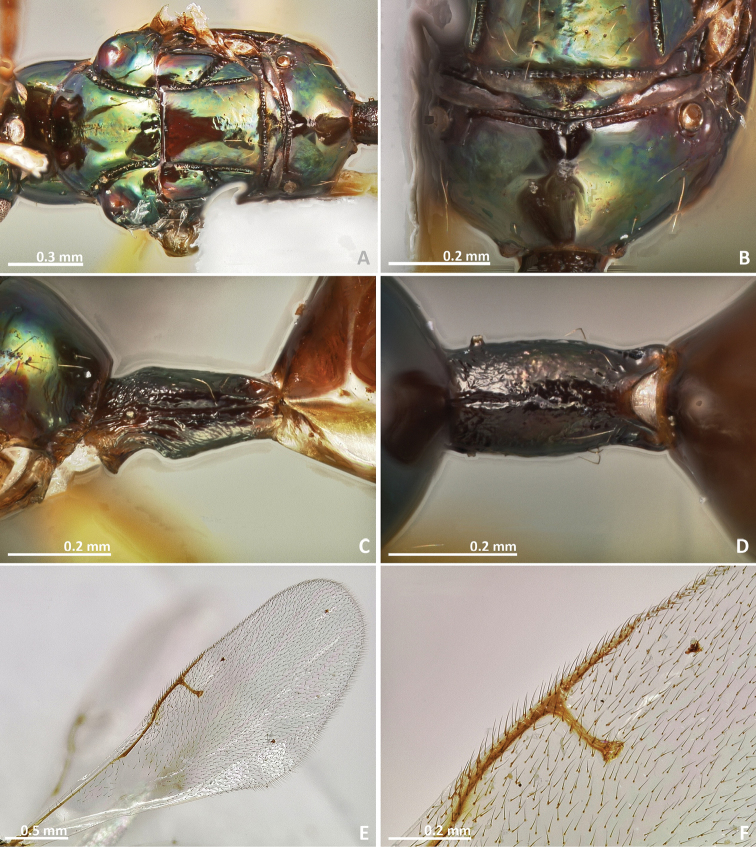
*Pseudidarnes laevis* sp. n. female. **A** mesosoma dorsal **B** propodeum dorsal **C** petiole lateral view **D** petiole dorsal view **E** wing **F** detail of venation.

*Male*. Body length 3 mm. Characters agreeing with the females, except the following. Body slender. Anelli more transverse. Ocelli slightly larger than the female’s ocelli. Wing more pilose.

**Figure 21. F21:**
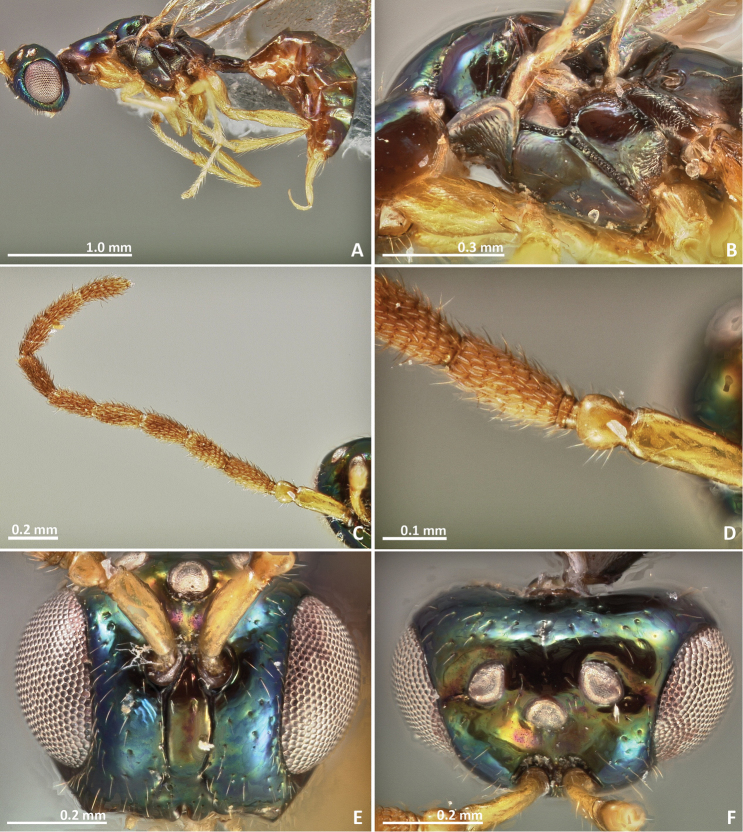
*Pseudidarnes laevis* sp. n. male. **A** habitus lateral **B** mesosoma lateral **C** antenna **D** anelli **E** head, anterior view **F** vertex, dorsal view.

**Figure 22.**
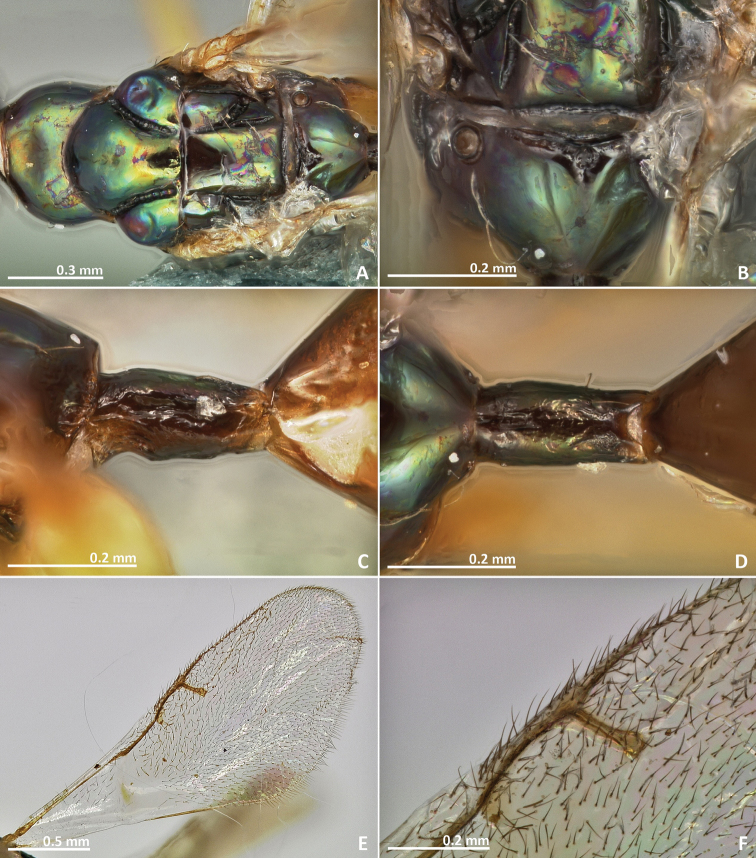
*Pseudidarnes laevis* sp. n. male. **A** mesosoma dorsal **B** propodeum dorsal **C** petiole lateral view **D** petiole dorsal view **E** wing **F** detail of venation.

##### Etymology.

The specific name refers to the smooth body sculpturation exhibited by this species.

##### Biology.

See *Pseudidarnes acaudus* biology section.

#### Pseudidarnes minerva

Girault, 1927

http://species-id.net/wiki/Pseudidarnes_minerva

**Figure 23.**
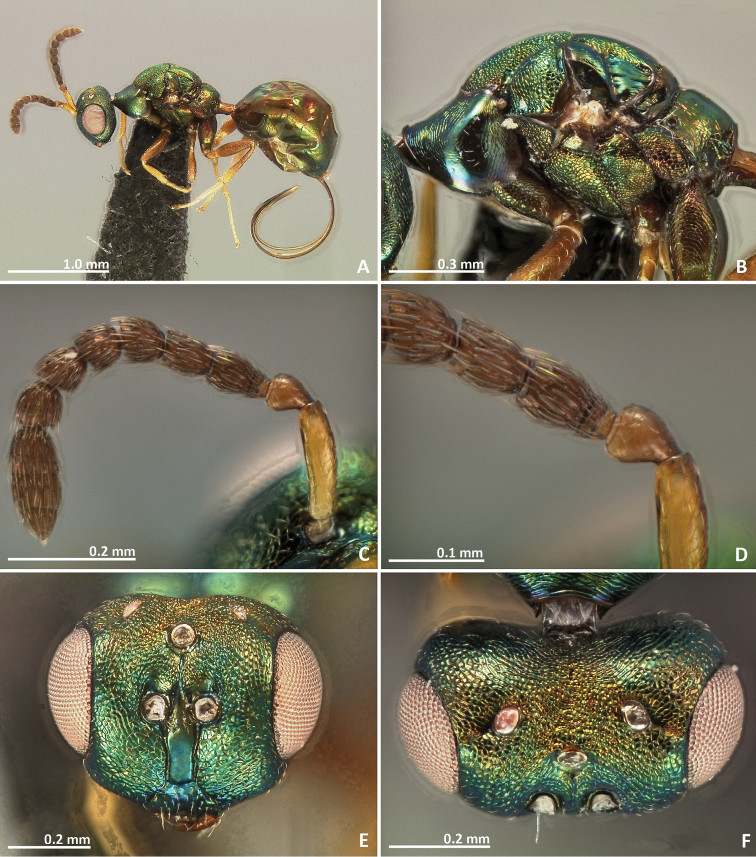
*Pseudidarnes minerva* Girault, 1927 female. **A** habitus lateral **B** mesosoma lateral **C** antenna **D** anelli **E** head, anterior view **F** vertex, dorsal view.

Figures 23 [Fig F24] [Fig F25] [Fig F26] –27

Pseudidarnes minerva ; Girault (1927) Records of the South Australian Museum. 3: 332. [description female]
Pseudidarnes minerva ; Bouček (1988) Australasian Chalcidoidea, Cap. 6 p. 159 (key), 187–188 pp. Figs 317–318. [New record, redescription]
Pseudidarnes minerva ; Fellowes et al. (1999) Behavioural Ecology and Sociobiology, 46: 95–102. [ecology]
Pseudidarnes minerva ; Early JW (2000) New Zealand Entomologist, 23: 29–30. Fig. 1. [natural history, description of wingless male, new occurrence]
Pseudidarnes minerva ; Cook (2005) In: Fellowes M, Holloway G, Rolff J (Eds), Insect Evolutionary Ecology, 83–110, Fig. 4.1. [ecology]
Pseudidarnes minerva ; Cruaud et al. (2011a) BMC Evolutionary Biology, 11: 15pp. [phylogenetic position]
Pseudidarnes minerva ; Cruaud et al. (2011b) Journal of Biogeography, 38: 209–225. [biogeography]

##### Material examined.

*Holotype*. ♀, **AUSTRALIA: Queensland:** Brisbane and Lake Manchester, -27.48°, 152.76°, 67m, [no date], ex *Ficus rubiginosa* (ASCI) [examined].

**AUSTRALIA: Queensland:** Amity, -27.39°, 153.44°, 5m, 7♀, 23.I.1999, Rasplus J.Y. & Meusnier, S, ex *Ficus rubiginosa*, n° JRAS00722 (CBGP), Ballina, -28.89°, 153.56°, 5m, 7♀, 3♂, 25.I.1999, Rasplus J.Y. & Meusnier, S, ex *Ficus rubiginosa*, n° JRAS00726 (CBGP), 1♀, 25.I.1999, Rasplus J.Y., ex *Ficus rubiginosa*, n° JRAS00727_01 (CBGP), Mount Molloy, -16.67°, 145.33°, 400m, 1♂, 25.X.2005, Jousselin E. & Coeur d’Acier A., ex *Ficus rubiginosa*, n° JRAS01418_28 (CBGP), Yungaburra, -17.27°, 145.58°, 700m, 5♀, 15.I.1999, Rasplus J.Y. & Meusnier, S, ex *Ficus rubiginosa*, n° JRAS00690 (CBGP); **Victoria:** Melbourne, -37.81°, 144.96°, 20m, 2♀, 2♂, I.1995, Cook J., ex *Ficus rubiginosa* (CBGP).

##### Diagnosis.

Mesosoma metallic green, mostly reticulate. Median sulcus of the propodeum extending over most of the propodeum length, not reaching the posterior margin. Postmarginal vein as long as the stigmal. Petiole longer than wide in dorsal view. Ovipositor sheaths longer than metasoma.

##### Description.

Body length 2.6 mm. Ovipositor sheaths length 2.33 mm.

*Coloration*. Metallic tinge present at least in some body regions. Predominantly green. Scape yellow. Petiole yellow brown. Flagellomeres brown. Coxae almost concolorous with mesosoma. Femora brown. Tibiae and tarsi predominantly yellow. Metatibia proximally browner. Petiole brown. Metasoma browner dorsally.

*Head*. Antennae inserted at or slightly below the middle line of compound eyes. Scape nearly 3 × as long as pedicel. Pedicel almost as long as wide, pyriform, and as long as first funicular segment. Anelli transverse. First funicular segment longer than wide to approximately as long as wide. Distal antennomeres forming a distinctive clava. Face sculpture reticulate. Face pilosity short and sparse. Supraclypeal area wide, its delimiting sulci not converging near epistomal groove, and its sculpture mostly smooth. Lateral ocelli nearly one diameter far from the eye margin.

*Mesosoma*. Pronotum short, nearly as long as high in lateral view, or slightly longer than high. Mesoscutum reticulate. Mesoscutellum engraved. Frenal sulcus densely crenulated. Mesepimeron sculpture reticulate. Metascutellum very short, inconspicuous, and almost completely covered by frenum. Propodeum with a carinulated longitudinal median line, extending over most of the propodeum length, not reaching the posterior margin. Propodeum sculpture engraved reticulate, smooth near the proximal region of median line of propodeum. Petiole 1.5 × as long as high in lateral view. Wings hyaline, with sparse pilosity. Marginal and postmarginal vein not particularly widened. Postmarginal vein as long as stigmal vein, or slightly shorter.

*Metasoma*. Petiole sculpture in lateral view slightly rugose. Petiole dorsally with a longitudinal median sulcus. Ovipositor sheaths long, distinctly protruding beyond metasoma apex. Ovipositor sheaths length 4.4 × hind tibia length, 0.9 × body length.

**Figure 24. F24:**
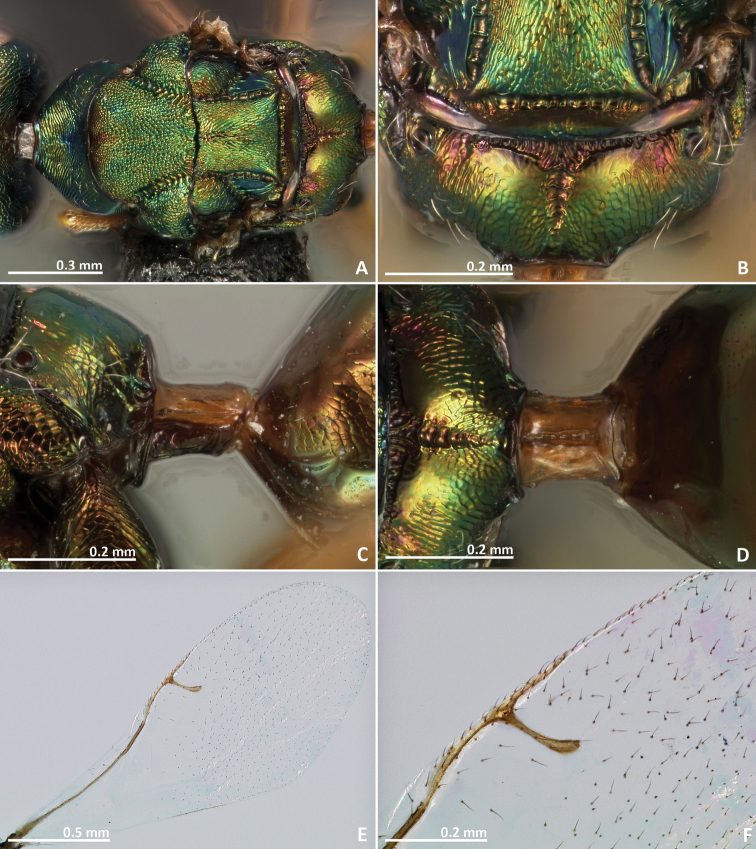
*Pseudidarnes minerva* Girault, 1927 female. **A** mesosoma dorsal **B** propodeum dorsal **C** petiole lateral view **D** petiole dorsal view **E** wing **F** detail of venation.

*Male*. Body length 2.3 mm. Characters agreeing with the females, except the following. body slender. Coxae brown, not concolorous with mesosoma. Anelli more transverse than female. Head sculpture engraved. Ocelli ca. 2 × the diameter of the female’s. Body sculpture fainter. Wing more pilose.

Wingless males were described for this species (Early 2000), but they are uncommon.

**Figure 25. F25:**
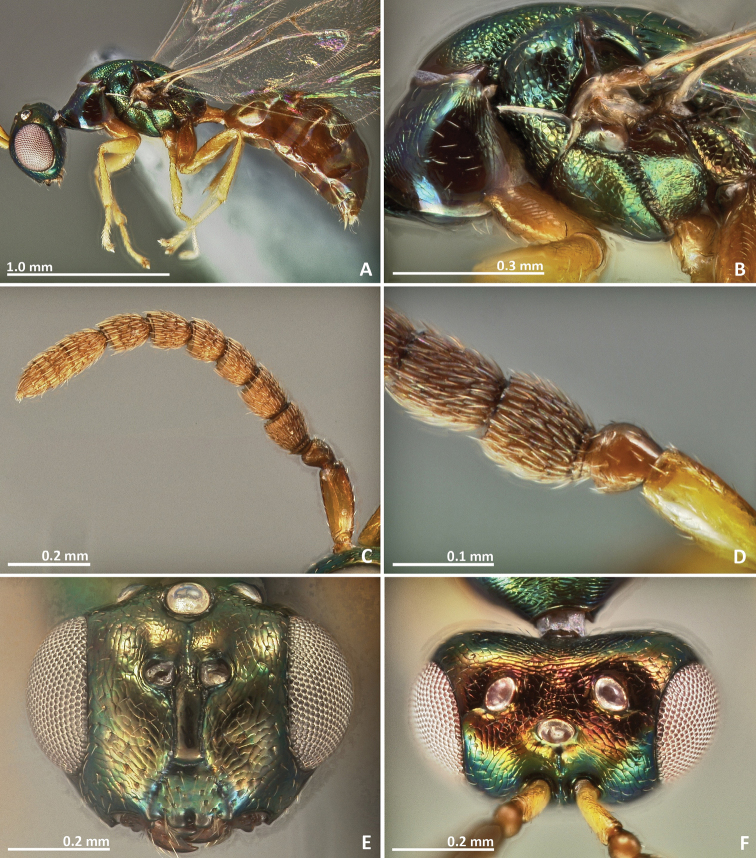
*Pseudidarnes minerva* Girault, 1927, male. **A** habitus lateral **B** mesosoma lateral **C** antenna **D** anelli **E** head, anterior view **F** vertex, dorsal view.

**Figure 26. F26:**
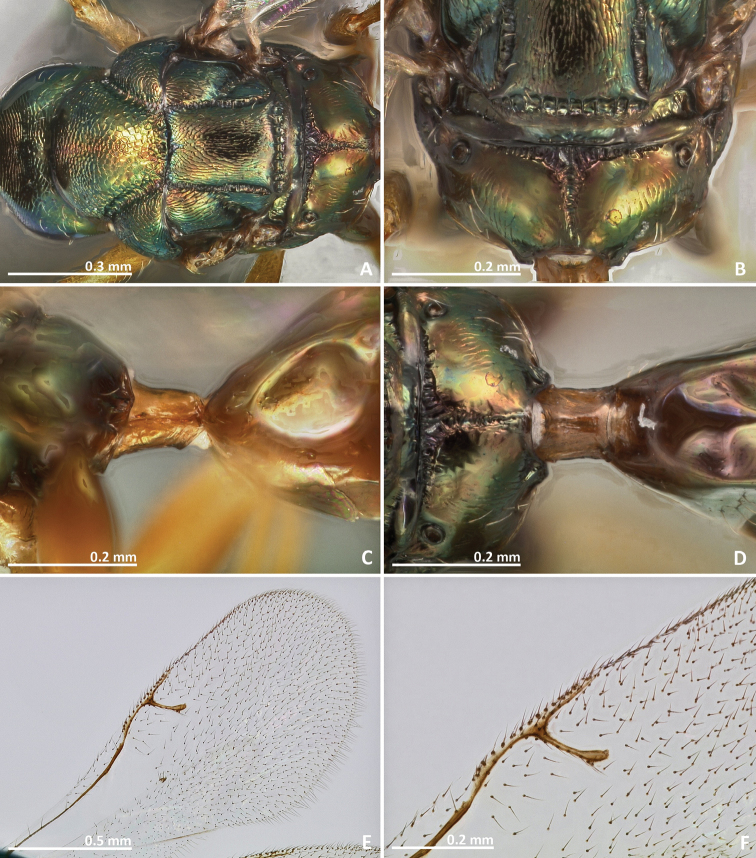
*Pseudidarnes minerva* Girault, 1927, male. **A** mesosoma dorsal **B** propodeum dorsal **C** petiole lateral view **D** petiole dorsal view **E** wing **F** detail of venation.

**Figure 27.**
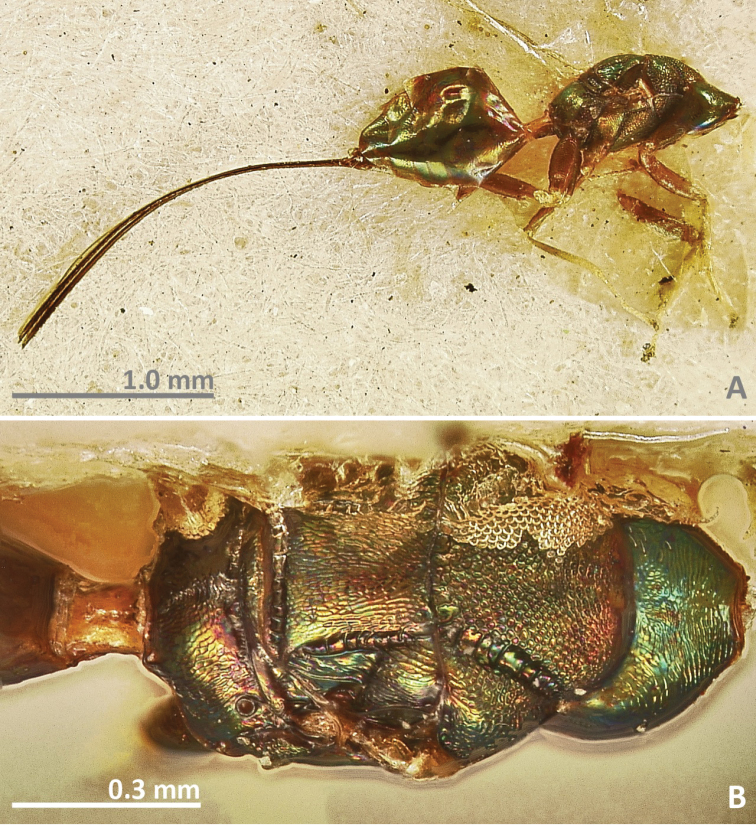
*Pseudidarnes minerva* Girault, 1927 holotype female. **A** habitus lateral **B** mesosoma in dorsal view.

##### Biology.

Reared from *Ficus rubiginosa* Desf. ex Vent. syconia. *Ficus rubiginosa* is pollinated by *Pleistodontes imperialis* Saunders. Usually collected in low abundances, but sometimes they are quite abundant, at least in Eastern Australia and Auckland (as seen by Early 2000). Details about the biology of *Pseudidarnes minerva* and other wasps associated with *Ficus rubiginosa* in New Zealand were described by Early (2000).

##### Molecular data.

GenBank sequences: COI HM770660; CytB HM770560; EF1a HM770504; rRNA 28S HM770665 (Cruaud et al. 2011a; Cruaud et al. 2011b).

#### Pseudidarnes sp. ex Ficus baola

Pseudidarnes sp. ex *Ficus baola*; Cruaud et al. (2011a) BMC Evolutionary Biology, 11: 15pp. [phylogenetic position]
Pseudidarnes sp. ex *Ficus baola*; Cruaud et al. (2011b) Journal of Biogeography, 38: 209–225. [biogeography]

##### Material examined.

**SOLOMON ISLANDS: Gatokae:** Mbulo island, -8.76°, 158.28°, 100m, 1♂, 20.II.2019, Cruaud A & Rasplus JY, ex *Ficus baola*, n°JRAS02523_02 (CBGP).

##### Biology.

Collected in *Ficus baola* C. C. Berg.

##### Comments.

Two specimens belonging to this species were included in the phylogenetic analysis by Cruaud et al. (2011a; 2011b). The remaining specimen is a male in poor state of conservation as it was removed from its gall before emergence, and cannot be described.

##### Molecular data.

GenBank sequences: COI HM770640, JN001572; Cytb HM770594; EF-1a HM770543; rRNA 28S HM770702 (Cruaud et al. 2011a; Cruaud et al. 2011b).

## Discussion

Due to their low abundance and relative rarity, some species were described here from very small series. Nevertheless, many specimens of *Pseudidarnes laevis* were collected from the same sample of figs, which indicates that they can sometimes show high infestation rates. This pattern is also observed in *Anidarnes*, which are also large gallers, and are usually found at low abundances (Bronstein 1999) but are more abundant in a few samples (personal observation, Farache et al. 2013). The relative low abundance (compared to fig pollinators and some other Sycophaginae that induce smaller galls) shown by *Pseudidarnes* and *Anidarnes* may explain the difficulty experienced by earlier taxonomists when studying and describing species belonging to these genera.

Here we collected and analysed wasps from both sexes in five from the eight studied species. *Pseudidarnes* males were similar to the females, in contrast to many other wasps associated with fig inflorescences, which are sexually dimorphic and show wingless males. Nevertheless, wingless males occur in very low abundance for *Pseudidarnes minerva* (Cook et al. 1997, Early 2000), so the fact that we did not find them in other *Pseudidarnes* species may be due to sampling effect, and therefore new dimorphic males may be found in the future. Also, wingless males do not leave their natal figs and remain within flowers and bracts, which may hamper their sampling by unaware collectors.

The eight *Pseudidarnes* species were collected from five different hosts. *Ficus xylosycia* hosted three species, namely *Pseudidarnes astridae*, *Pseudidarnes badiogeminus*, and *Pseudidarnes flavicollis*. The former two species were reared together in the same sample. An undetermined fig species also hosted two species, *Pseudidarnes laevis* and *Pseudidarnes acaudus* (reared in a same sample). Despite the fact that more than one species may share the same fig, we did not find any *Pseudidarnes* species occurring in more than one host, which possibly indicates that they are host specific.

*Pseudidarnes acaudus* is the most divergent of all collected species, and is easily recognizable by its extremely short ovipositor sheaths. The other species can be separated into two morphological groups that correspond well to their geography and to their host association. Papuan species show a slender mesosoma, long funicular segments, and body sculpture that is mostly smooth, while Australian species have a short and robust mesosoma, shorter funicular segments, and a reticulate body sculpture. The taxonomy of the section *Malvanthera* is also geographically consistent since two of its subsections (namely *Malvantherae* and *Platypodeae*) are primarily Australian, while subsection *Hesperidiiformes* has its diversity centre in New Guinea (Rønsted et al. 2008). All Australian hosts collected belong to subsection *Platypodeae*, whereas *Ficus xylosycia* from Papua belongs to subsect. *Hesperidiiformes*. The host of *Pseudidarnes laevis* is unknown, but probably it belongs to subsect. *Hesperidiiformes*, since the only species belonging to other *Malvanthera* subsections known to occur in Papua is *Ficus obliqua* (Rønsted et al. 2008).

This is the first revisionary treatment of *Pseudidarnes*. We believe that, due to the lack of previous careful sampling, several *Pseudidarnes* species remain to be discovered especially in New Guinea, but also in Australia. We hope that this work will encourage discovery and further studies on the biology of *Pseudidarnes* species.

## Supplementary Material

XML Treatment for Pseudidarnes

XML Treatment for Pseudidarnes acaudus

XML Treatment for Pseudidarnes astridae

XML Treatment for Pseudidarnes badiogeminus

XML Treatment for Pseudidarnes cooki

XML Treatment for Pseudidarnes flavicollis

XML Treatment for Pseudidarnes kjellbergi

XML Treatment for Pseudidarnes laevis

XML Treatment for Pseudidarnes minerva

XML Treatment for Pseudidarnes sp. ex Ficus baola
